# Inheritance pattern of tetraploids pummelo, mandarin, and their interspecific hybrid sour orange is highly influenced by their phylogenomic structure

**DOI:** 10.3389/fpls.2023.1327872

**Published:** 2023-12-08

**Authors:** Pablo Aleza, Miguel Fernando Garavello, Houssem Rouiss, Ana Cristina Benedict, Andres Garcia-Lor, Maria Hernández, Luis Navarro, Patrick Ollitrault

**Affiliations:** ^1^ Departamento de Citricultura y Producción Vegetal, Instituto Valenciano de Investigaciones Agrarias (IVIA), Moncada, Valencia, Spain; ^2^ Concordia Agricultural Experimental Station, National Agricultural Technology Institute, Concordia, Entre Ríos, Argentina; ^3^ Centre de coopération internationale en recherche agronomique pour le développement Centre de coopération internationale en recherche agronomique pour le développement (CIRAD), Amélioration Génétique et Adaptation des Plantes Méditerranéennes et Tropicales (UMR AGAP) Institut, Montpellier, France; ^4^ AGAP Institut, Univ Montpellier, Centre de coopération internationale en recherche agronomique pour le développement (CIRAD), INRAE, Institut Agro, Montpellier, France

**Keywords:** citrus, triploid, tetrasomic, disomic, SSR and SNP markers, seedless, rootstock, breeding

## Abstract

Citrus polyploidy is associated with a wide range of morphological, genetic, and physiological changes that are often advantageous for breeding. Citrus triploid hybrids are very interesting as new seedless varieties. However, tetraploid rootstocks promote adaptation to different abiotic stresses and promote resilience. Triploid and tetraploid hybrids can be obtained through sexual hybridizations using tetraploid parents (2x × 4x, 4x × 2x, or 4x × 4x), but more knowledge is needed about the inheritance pattern of tetraploid parents to optimize the efficiency of triploid varieties and tetraploid rootstock breeding strategies. In this work, we have analyzed the inheritance pattern of three tetraploid genotypes: ‘Chandler’ pummelo (*Citrus maxima*) and ‘Cleopatra’ mandarin (*Citrus reticulata*), which represent two clear examples of autotetraploid plants constituted by the genome of a single species, and the ‘Sevillano’ sour orange, which is an allotetraploid interspecific hybrid between *C. maxima* and *C. reticulata*. Polymorphic simple sequence repeat (SSR) and single-nucleotide polymorphism (SNP) markers were used to estimate parental heterozygosity restitution, and allele frequencies for centromeric loci were used to calculate the preferential pairing rate related to the proportion of disomic and tetrasomic segregation. The tetraploid pummelo and mandarin displayed tetrasomic segregation. Sour orange evidenced a clear intermediate inheritance for five of the nine chromosomes (1, 2, 5, 7, and 8), a slight tendency toward tetrasomic inheritance on chromosome 3, and intermediate inheritance with a tendency toward disomy for chromosomes 4, 6, and 9. These results indicate that the interspecific versus intraspecific phylogenomic origin affects preferential pairing and, therefore, the inheritance patterns. Despite its high level of heterozygosity, the important preferential chromosome pairing observed in sour orange results in a limited diversity of the genotypic variability of its diploid gametes, and consequently, a large part of the genetic value of the original diploid sour orange is transferred to the tetraploid progenies.

## Introduction

The hypothesis that polyploidy can facilitate adaptation to both abiotic and biotic stresses and that whole genome duplication can act as a buffer to mitigate their effects is increasingly gaining support (Van de Peer et al., 2021). Today, polyploidy is considered a frequent mode of speciation with long-term ecological and evolutionary consequences in plants. Polyploid plants are more resilient to extreme environments due to their increased genetic variation and the buffering effect of duplicated genes (Van de Peer et al., 2021). Citrus species are subjected to adverse environmental conditions throughout their entire life cycle, as well as over consecutive cropping seasons. *Citrus* and related genera of Aurantioideae are generally diploid (Krug, 1943) (2n = 2x = 18), but some higher euploid genotypes are extant in the citrus germplasm. The most common euploid variations are triploids and tetraploids (Lee, 1988; Ollitrault et al., 2020). Citrus polyploidy is often associated with a wide range of morphological and physiological changes that are often advantageous under adverse environmental conditions (De Ollas et al., 2019). It has been shown that tetraploid rootstocks promote adaptation to different abiotic stresses and promote resilience (Ruiz et al., 2020). They have been described as having better tolerance to cold, salinity, and drought than their diploid counterparts (Allario et al., 2013; Ruiz et al., 2016a; Oustric et al., 2017). Oustric et al. (2019) and Ruiz et al. (2016b) indicated that tetraploid rootstocks are less affected by nutritional stress and less sensitive to boron excess than the corresponding diploid rootstocks. Recently, it has also been shown that triploid hybrids have improved tolerance to low temperatures as compared to diploids and also display highly volatile organic compounds involved in oxidative stress protection (Lourkisti et al., 2020). Similarly, Lourkisti et al. (2021) showed that triploid hybrids enhance the recovery capacity after a water deficit; this improved response can be attributed to changes in their morphological and cytological structure that provide more energy to adapt to adverse environments (Lourkisti et al., 2022). Additionally, triploid hybrids have very low gametic fertility and generally do not produce seeds nor induce seeds in other varieties by cross-pollination. Thus, triploid hybrids are very interesting for selecting new seedless varieties. Moreover, the parthenocarpy of triploid varieties makes them useful for beekeeping due to their compatibility with the presence of bees in citrus plants, without the risk of producing highly seedy fruits. This is of great importance today since bee populations have dramatically declined in occurrence and diversity in the USA and Europe (Gurung and Chettri, 2022), making triploid varieties a bee-friendly option. Considering these combined characteristics, polyploidy breeding appears to be a relevant strategy for developing both new triploid seedless varieties, which are more respectful to the environment, and tetraploid rootstocks with enhanced abiotic stress tolerance.

Citrus polyploidy results from somatic or sexual polyploidization and offers opportunities in breeding programs. Adventitious embryony from nucellar cells is the origin of apomixis in citrus, and spontaneous duplication of chromosomes in nucellar cells results in the production of tetraploid seedlings (Aleza et al., 2011). Additionally, artificial tetraploid plants can be obtained by somatic hybridization using protoplast fusion (Dambier et al., 2011; Grosser and Gmitter, 2011), as well as with antimitotic chemicals such as colchicine and oryzalin (Aleza et al., 2009). Both tetraploid plants resulting from chromosome doubling in nucellar cells or antimitotic treatments are doubled diploid (DD), with two times the original chromosome haplotypes of their parental diploids. Triploid hybrids can be recovered by sexual hybridizations with diploid parents *via* unreduced gamete formation (Cuenca et al., 2011; Cuenca et al., 2015) or by using tetraploid plants as male or female parents (Aleza et al., 2012a; Aleza et al., 2012b; Ahmed et al., 2020). Tetraploid hybrids can be produced with high efficiency in 4x × 4x sexual hybridizations (Grosser and Gmitter, 2011; Calvez et al., 2023), although tetraploid hybrids may also occur to a lesser extent in 2x × 4x or 4x × 2x due to unreduced male or female gametes (Aleza et al., 2012a; Rouiss et al., 2017).

The meiotic behavior of tetraploid parents has a strong impact on the genetic diversity of the population of polyploid hybrids and their breeding efficiency. The genetic structure of diploid gamete populations produced by 4x parents and particularly parental heterozygosity restitution (PHR) depends on preferential chromosome pairing (PP), as well as on the double reduction (DR) rate, with two extreme models: disomic inheritance in allotetraploids and tetrasomic in autotetraploids (Stift et al., 2008). Allotetraploids combine the genomes of two different species and present two sets of homoeologus chromosomes, each consisting of two homologous chromosomes. Disomic inheritance occurs when a chromosome pairs exclusively with its homolog and only bivalents are formed, with the transmission of 100% of the interspecific heterozygosity by each gamete. In contrast, the four homologous chromosomes in the autotetraploids, resulting from polyploidization in a single species, have the same opportunity to mate during meiosis, leading to tetrasomic inheritance with potential multivalent formation. For autotetraploids, this hypothetically leads to between 55% and 66% of PHR, depending on the DR rate (Muller, 1914; Mather, 1936). DR results in two sister chromatids being recovered in a single gamete, leading to a PHR decrease. In cases where parents are divergent but have retained sufficient homology to prevent exclusive PP, intermediate inheritance patterns between disomic and tetrasomic can be expected (Stift et al., 2008; Aleza et al., 2016). Stift et al. (2008) developed a likelihood-based approach to evaluate whether disomic, intermediate, or tetrasomic inheritances best fit the segregation of genetic markers and to estimate PP and DR. This method was simplified afterward by Aleza et al. (2016) for doubled diploids originating from spontaneous or chemically induced somatic chromosome doubling.

Molecular studies and recent genomic studies (Wu et al., 2014; Curk et al., 2016; Oueslati et al., 2017; Wu et al., 2018; Ahmed et al., 2019) have provided a clear understanding of the evolution of cultivated *Citrus*, revealing the existence of four ancestral taxa as the ancestors of most of the cultivated citrus: [*Citrus maxima* (Burm.) Merr., pummelos; *Citrus medica* L., citrons; *Citrus reticulata* Blanco, mandarins; and *Citrus micrantha* Wester, a wild papeda species]. The current cultivated secondary species [*Citrus aurantium* L., sour orange; *Citrus sinensis* (L.) Osbeck, sweet orange; *Citrus paradisi* Macf., grapefruit; *Citrus limon* (L.) Burm. F., lemon; and *Citrus aurantifolia* (Christm.) Swingle, lime] and modern cultivars are the result of admixture between these ancestral taxa. Previous works (Aleza et al., 2016; Kamiri et al., 2018; Rouiss et al., 2018; Ahmed et al., 2020; Calvez et al., 2020; Calvez et al., 2023) have revealed the great impact of the phylogenomic structure of tetraploid parents on the meiotic behavior and, therefore, on the genotypic variability of diploid gamete populations. According to the admixture level of tetraploid parents, these authors have observed tetrasomic to disomic inheritance, with frequent situations of intermediate inheritance. In the present work, we have analyzed the inheritance pattern of the tetraploids ‘Sevillano’ sour orange, ‘Chandler’ pummelo, and ‘Cleopatra’ mandarin. All are doubled diploids resulting from somatic chromosome doubling of the corresponding diploid varieties. These three tetraploid genotypes have been selected since two of them, ‘Chandler’ pummelo and ‘Cleopatra’ mandarin, represent two ancestral species, *C. maxima* and *C. reticulata*, respectively, whereas ‘Sevillano’ sour orange is an F1 direct hybrid between these two ancestral species (Wu et al., 2014). The tetraploid genotypes of ‘Chandler’ pummelo and ‘Cleopatra’ mandarin represent two clear examples of autotetraploid plants, as they constitute the genome of a single species, while the ‘Sevillano’ sour orange can be considered as an allotetraploid interspecific hybrid between *C. maxima* and *C. reticulata*. Therefore, these three genotypes are of great interest for studying how phylogenomic structure affects the genotypic structure of diploid gamete populations, their inheritance pattern, and their implications on polyploid citrus breeding programs based on sexual hybridizations.

## Materials and methods

### Plant material

Citrus triploid hybrids were obtained from 2x × 4x sexual hybridizations using diploid clementine (*Citrus clementine* Hort. Ex Tan.) as the female parent with the tetraploid genotypes ‘Sevillano’ sour orange, ‘Cleopatra’ mandarin, and ‘Chandler’ pummelo as male parents. The tetraploid ‘Chandler’ pummelo was obtained by shoot-tip grafting *in vitro* combined with colchicine treatment (Aleza et al., 2009), whereas ‘Sevillano’ sour orange and ‘Cleopatra’ mandarin were recovered from the spontaneous chromosome doubling of nucellar cells (Aleza et al., 2011). Pollen fertility of parents at diploid and tetraploid ploidy levels was analyzed by *in vitro* germination test according to the methodology described by Lora et al. (2022). The percentage of pollen grain germination was 39% and 26% for 2x and 4x ‘Sevillano’ sour orange, respectively; 56% and 51% for 2x and 4x ‘Cleopatra’ mandarin, respectively; and 92% and 74% for 2x and 4x ‘Chandler’ pummelo, respectively. Triploid hybrids were produced following the methodology described by Aleza et al. (2012a). After embryo rescue, the ploidy of the obtained plantlets was analyzed by flow cytometry according to Aleza et al. (2012a). All plantlets were triploids. In total, 87, 84, and 75 triploid hybrids were recovered from pollination with tetraploid ‘Sevillano’ sour orange, ‘Cleopatra’ mandarin, and ‘Chandler’ pummelo, respectively.

### Simple sequence repeat and single-nucleotide polymorphism marker analysis for triploid progeny genotyping

The parents were genotyped using a total of 77 simple sequence repeat (SSR) and single-nucleotide polymorphism (SNP) markers displaying a homogenous distribution in the nine chromosomes (CHRs) of the reference genetic map of clementine (Ollitrault et al., 2012b). For each tetraploid parent, we selected the markers that proved heterozygosity and polymorphism with clementine to perform genotyping of the corresponding triploid progenies.

#### SSR markers

A total of 55 SSR markers were analyzed (**Additional Table 1**). PCR amplifications were performed using a Thermocycle rep gradient S (Eppendorf^®^) in 15 µL final volume containing 0.8 U of Taq DNA polymerase (Fermentas^®^), 2 ng/mL of citrus DNA, 0.2 mM of wellRED (Sigma^®^) dye-labeled forward primer, 0.2 mM of non-dye-labeled reverse primer, 0.2 mM of each dNTP, 10X PCR buffer, and 1.5 mM MgCl_2_. The PCR protocol was as follows: denaturation at 94°C for 5 min followed by 40 repeats of 30 s at 94°C, 1 min at 50°C or 55°C, 45 s at 72°C, and a final elongation step of 4 min at 72°C. Capillary electrophoresis was carried out using a CEQ™ 8000 Genetic Analysis System (Beckman Coulter Inc., Brea, CA, USA). PCR products were initially denatured at 90°C for 2 min, injected at 2 kV for 30 s, and subsequently separated at 6 kV for 35 min. Alleles were sized, based on a DNA size standard (400 bp). The GenomeLab™ GeXP v.10.0 genetic analysis software was used for data collection. Allele dosage was calculated using the MAC-PR (microsatellite DNA allele counting-peak ratio) method (Esselink et al., 2004), which was validated in citrus by Cuenca et al. (2011).

#### SNP markers

Twenty-two SNP markers were genotyped (**Additional Table 1**) using KASPar technology by LGC Genomics (http://www.lgcgenomics.com). Of the total of SNP markers used, 13 were new (**Table 1**) and were developed from a genotyping-by-sequencing (GBS) diversity analysis (unpublished data). The KASPar™ genotyping system is a competitive, allele-specific dual Förster Resonance Energy Transfer (FRET)-based assay for SNP genotyping. Primers were directly designed by LGC Genomics Company based on the SNP locus flanking sequence (approximately 50 nt on each side of the SNP). SNP genotyping was performed using the KASPar technique. A detailed explanation of the specific conditions and reagents can be found in Cuppen (2007). The allele doses in heterozygous triploid hybrids were identified from the relative allele signals as described by Cuenca et al. (2013).

**Table 1:** Primer sequences of the new SNP markers developed in this paper for use in KASPar™ assay.

Markers name	SNP-specific primer		Common primer
1_14053816	Allele X:	TGCTTACAGAAACATTCGAGTTGGC	GTATCCCAAATTTGAGCTTTGATGAGCTT
	Allele Y:	TTGCTTACAGAAACATTCGAGTTGGG	
2_60291	Allele X:	CTGATAGACGCAGTGAGCAGGTA	CCAGCAATTGGGGAACACCTGAATT
	Allele Y:	TGATAGACGCAGTGAGCAGGTG	
2_36363713	Allele X:	AAAATAAATCCACAATGTTAATGTTGCTGG	CAAGTGAGTCCAACTTCATAAATTGCTCAT
	Allele Y:	AAAATAAATCCACAATGTTAATGTTGCTGC	
3_17626	Allele X:	CTCATGTAAATACTCAATTGCTTGCGC	CGCATGGCTATAGCCTTCCAACTT
	Allele Y:	TCTCATGTAAATACTCAATTGCTTGCGA	
3_51024515	Allele X:	ATTGGAGAAGCCGAGAAGGCGA	CAGCTCAGCTCACGTGATATTTCTAATTA
	Allele Y:	GGATTGGAGAAGCCGAGAAGGCGG	
5_27500244	Allele X:	GTTTATTTGATGTTAACTTGAGTATCTAAAAG	CTTCTTCACACTAAGAAATAGAACTCTCAT
	Allele Y:	GTTTATTTGATGTTAACTTGAGTATCTAAAAC	
6_5996116	Allele X:	ATGAAGACTAGAAGCTTAAAGTTAGATGAT	GCTCAGCAGGTGCAGTTCCACTT
	Allele Y:	ATGAAGACTAGAAGCTTAAAGTTAGATGAA	
7_20255	Allele X:	CCCAGCAAAAACACTTCTTGTTGTTTTA	CTGGGCCTGGCTTTCAGTCTCT
	Allele Y:	CCAGCAAAAACACTTCTTGTTGTTTTG	
7_15708026	Allele X:	GCTTACAACCCTTTAAACTCAGTTACGT	GGACAGGCAAGAAAAAGGCTAGGAA
	Allele Y:	TTACAACCCTTTAAACTCAGTTACGC	
8_15415145	Allele X:	AAGGCCAGAATCAACGGAGTTTC	ACCGTCCGCTATGACCTCCCTA
	Allele Y:	GAAGGCCAGAATCAACGGAGTTTA	
8_25026006	Allele X:	GCTACACACAGAAAGAGAGAGAGAG	GAGAGTTAAAGTCGGATAGATGAAAGATAA
	Allele Y:	GCTACACACAGAAAGAGAGAGAGAA	
9_26016472	Allele X:	GAAAACTCAATTTACCAATAAGTGCTCG	CGGCTAAGGAGCATCAATTGACCAT
	Allele Y:	CGAAAACTCAATTTACCAATAAGTGCTCT	
9_31288895	Allele X:	GCGCAGAAATAATTCCCTATACTACAAA	CCGTTGGCCCCTTTTTCTTCGTAAA
	Allele Y:	CGCAGAAATAATTCCCTATACTACAAG	

SNP, single-nucleotide polymorphism.

## Data analysis

### Confirmation of the parent producing the diploid gamete and inference of the diploid gamete genotype

For each triploid hybrid, it was verified that the diploid gametes were transmitted from the tetraploid parent. Markers with total differentiation between the parents (A_1_A_2_ × A_3_A_3_A_3_A_3_ and A_1_A_2_ × A_3_A_3_A_4_A_4_) were used for this purpose as described in Aleza et al. (2016). Once the parental origin of the diploid gamete was confirmed, the inference of the allelic configurations of the diploid gametes from triploid hybrid genotyping was carried out. For a locus bearing completely different parental allelic configurations (A_1_A_2_ × A_3_A_3_A_4_A_4_), the genotype of the diploid gamete was read directly from the triploid hybrid structure. When the male and female parents shared one allele (A_1_A_2_ × A_2_A_2_A_2_A_2_ and A_1_A_2_ × A_2_A_2_A_3_A_3_), the inference of the diploid male gamete structure for the triploid hybrids that had inherited the common allele from the female parent was performed from the estimated allele dosage in the triploid hybrid.

### Parental heterozygosity restitution and expected heterozygosity

For each locus, the PHR was calculated as the percentage of individuals with the same heterozygous allelic configuration as the tetraploid male parent. As we selected heterozygous markers for tetraploid parents, PHR was equivalent to the observed heterozygosity in each diploid gamete population.

### Analysis of the deviation from expected allelic and gametic segregation under a tetrasomic model

The potential distortion in allelic segregation on the diploid gamete populations was analyzed using a chi-squared test (χ^2^) of conformity with the theoretical frequencies A_1_ = 0.5 and A_2_ = 0.5.

Genotypic data of centromeric loci were used to study the deviation from the expected gametic segregation under a tetrasomic model without DR for each tetraploid parent. The p-values for the chi-squared test according to the tetrasomic theoretical frequency for each possible gamete (for a duplex locus A_1_A_1_A_2_A_2_: A_1_A_1_ = 1/6, A_2_A_2_ = 1/6, and A_1_A_2_ = 4/6) were computed using Microsoft Excel (Microsoft Corporation, 2018. Microsoft Excel, https://office.microsoft.com/excel). In the case of a significant p-value (threshold = 0.01) for this first model, a second conformity test was performed according to the tetrasomic model with maximum equational chromatid segregation (Mather, 1936; Marsden et al., 1987) with theoretical frequencies: A_1_A_1_ = 2/9, A_2_A_2_ = 2/9, and A_1_A_2_ = 5/9. To limit the false discovery rate (FDR) in multiple testing, the Benjamini–Hochberg correction for multiple comparisons (Benjamini and Hochberg, 1995) was then applied with a q-value threshold of 0.01 for both allelic and genotypic data.

### Estimation of preferential association frequency and maximum double reduction rate

Stift et al. (2008) proposed a segregation model for allotetraploid citrus hybrids, which was simplified by Aleza et al. (2016) for tetraploids resulting from somatic chromosome doubling. It is considered that in such tetraploids, the expected frequencies of each type of gamete for centromeric loci depend only on the “tetrasomic” parameter (τ), corresponding to the proportion of gametes formed by random associations of meiotic chromosomes (i.e., random bivalent or tetravalent pairing). The estimation of τ was performed using a maximum likelihood approach from the analysis of the marker closest to the centromere for each chromosome, according to Aleza et al. (2016). The considered positions of the centromere were those proposed by Aleza et al. (2014) for each chromosome of the clementine genetic map (Ollitrault et al., 2012b). Confidence intervals (CIs) were estimated following a similar approach to the logarithm of the odds (LOD) drop-off method (Lander and Botstein, 1989) by finding the values at either side of the estimated τ that corresponded to a 10-fold decrease in probability. Then, PP was calculated as 1 − τ. PP values ranged from 1 for full disomy to 0 for complete tetrasomic inheritance. The DR rate and its CI for each chromosome were estimated as proposed by Aleza et al. (2016). Briefly, DR was estimated from τ values for each chromosome for the markers furthest from the centromere applying a maximum likelihood approach, and the CI corresponded to the values on each side with a 10-fold decrease in the probability.

### Population diversity organization

Population diversity organization was studied using DARwin6 software (Perrier and Jacquemoud-Collet, 2021) by neighbor-joining analysis using the simple matching dissimilarity index (*d_i−j_*) between pairs of loci (units):


$$d_{i-j}=1-\frac{1}{L}\sum_{l=1}^{L}\frac{m_{l}}{\pi },$$

where $\;d_{i-j}$ is the dissimilarity between units *i* and *j*, *L* is the number of loci, and $m_{l}$ is the number of matching alleles for locus *l*. From the dissimilarity matrix obtained, a factorial analysis was computed.

## Results and discussion

### Parental origin of the diploid gametes

The genotyping of clementine and the tetraploid parents allowed for the identification of markers for each progeny in heterozygosity for the tetraploid parents with different profiles in clementine. Thirty markers were selected for the progeny recovered with 4x ‘Sevillano’ sour orange. It included eight SNPs and 22 SSR markers, of which 12 SSR markers (CiBE5720, JK-taa15, mCrCIR03C08, JK-TAA41, MEST256, mCrCIR04E02, mCrCIR03G05, MEST104, mCrCIR01F04a, mCrCIR02G02, mCrCIR02A09, and mCrCIR07F11) displayed full allelic differentiation between clementine and sour orange. Twenty-seven SSR markers were used for the progeny obtained with 4x ‘Cleopatra’ mandarin. Of these markers, six (mCrCIR02G08, mCrCIR03C08, CF-CA31, MEST132, CIBE5866, and mCrCIR01F04a) showed unambiguous allelic differentiation between parents. Regarding the last progeny produced with 4x ‘Chandler’ pummelo, 36 markers were selected: 12 SNPs and 24 SSR markers, of which 12 (CI01C07, CMS30, MEST56, CIBE4818, mCrCIR03B07, mCrCIR01F04a, mCrCIR07B05, MEST830, mCrCIR02C09, MEST330, mCrCIR07F11, and MEST308) presented full allelic differentiation between clementine and ‘Chandler’ pummelo. For each of these SSR markers with unambiguous allelic differentiation between parents, and each progeny, all triploid hybrids showed triallelic configuration, and the tetraploid parent was confirmed to be the origin of the diploid gamete, as we expected. For the remaining molecular markers with biallelic configuration, their genetic configurations were inferred for all marker–gamete combinations by allele dosage calculation according to Cuenca et al. (2013) for SNP markers and Cuenca et al. (2015) for SSR markers (**Additional Tables 2**-**4**).

Potential allelic segregation distortion in the three diploid gamete populations was tested at each marker using chi-squared analysis applying the Benjamini–Hochberg correction for multiple comparisons to evaluate the q-values with a 0.01 threshold (**Additional Table 5**). No significant distortion was observed for ‘Chandler’ pummelo, ‘Cleopatra’ mandarin, and ‘Sevillano’ sour orange gametes.

### Parental heterozygosity restitution and genetic distance of diploid gametes produced by ‘Sevillano’ sour orange, ‘Cleopatra’ mandarin, and ‘Chandler’ pummelo tetraploid genotypes

#### Variation of PHR

**Figure 1:**
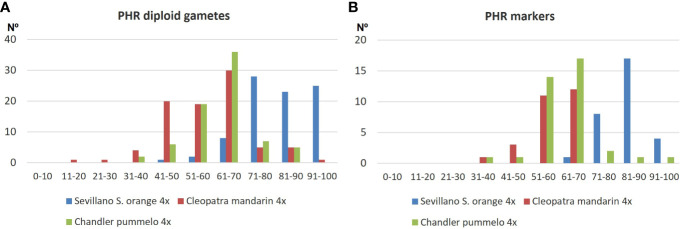
Distribution of PHR at the gamete **(A)** and marker **(B)** level in the diploid gamete population obtained by tetraploid sour orange, mandarin, and pummelo. PHR, parental heterozygosity restitution.

Doubled diploid ‘Cleopatra’ mandarin and ‘Chandler’ pummelo displayed unimodal PHR distributions with a mode between 0.61% and 0.70% (**Figure 1A**), whereas doubled diploid ‘Sevillano’ sour orange showed a unimodal distribution with negative asymmetry, producing 25 diploid gametes with PHR values greater than 90% (**Figure 1A**). Regarding PHR distribution for the markers, the mode was also between 0.61 and 0.70 for ‘Cleopatra’ mandarin and ‘Chandler’ pummelo, while it was between 0.81 and 0.90 for ‘Sevillano’ sour orange (**Figure 1B**).

The values of PHR obtained for each molecular marker and genotype are indicated in **Additional Table 5**, whereas **Table 2** displays the average values of PHR for each chromosome and the whole population of diploid gametes. ‘Chandler’ pummelo displayed an average value of PHR for the whole population of 0.619 ± 0.088, slightly higher than that obtained for ‘Cleopatra’ mandarin (0.583 ± 0.069), but clearly lower than that of ‘Sevillano’ sour orange (0.837 ± 0.060). It is important to note that, whereas for ‘Cleopatra’ mandarin and ‘Chandler’ pummelo the average value of PHR for each chromosome was similar, a clear difference was obtained when compared with ‘Sevillano’ sour orange, suggesting a different inheritance pattern (**Table 2**).

**Table 2:** Parental heterozygosity restitution and genetic distance of diploid gametes produced by ‘Sevillano’ sour orange, ‘Cleopatra’ mandarin, and ‘Chandler’ pummelo tetraploid genotypes.

CHR	Tetraploid ‘Sevillano’ sour orange				Tetraploid ‘Cleopatra’ mandarin				Tetraploid ‘Chandler’ pummelo			
	PHR	PHR SD	Av D	Av D CI	PHR	PHR SD	Av D	Av D CI	PHR	PHR SD	Av D	Av D CI
1	0.853	0.029	0.138	0.006	0.576	0.048	0.337	0.007	0.631	0.047	0.298	0.007
2	0.755	0.053	0.212	0.006	0.529	0.071	0.362	0.006	0.602	0.013	0.322	0.007
3	0.770	0.024	0.205	0.006	0.613	0.042	0.283	0.006	0.669	0.083	0.267	0.005
4	0.910	0.010	0.089	0.005	0.656	0.036	0.296	0.007	0.569	0.078	0.339	0.008
5	0.810	0.017	0.171	0.007	0.607	0.024	0.319	0.006	0.636	0.039	0.298	0.008
6	0.888	0.040	0.108	0.005	0.488	0.102	0.381	0.008	0.613	0.038	0.307	0.007
7	0.845	0.021	0.143	0.007	0.573	0.083	0.322	0.007	0.573	0.034	0.338	0.007
8	0.883	0.021	0.112	0.006	0.618	0.036	0.305	0.007	0.675	0.132	0.266	0.006
9	0.850	0.036	0.141	0.006	0.596	0.047	0.324	0.007	0.598	0.139	0.308	0.006
Total	0.837	0.060	0.149	0.002	0.583	0.069	0.327	0.003	0.619	0.088	0.303	0.002

PHR, parental heterozygosity restitution; PHR S D, PHR standard deviation; Av D, average genetic distance; Av D CI, average genetic distance confidence interval α 0.05; CHR, chromosome.

#### Genotypic diversity organization

**Figure 2:**
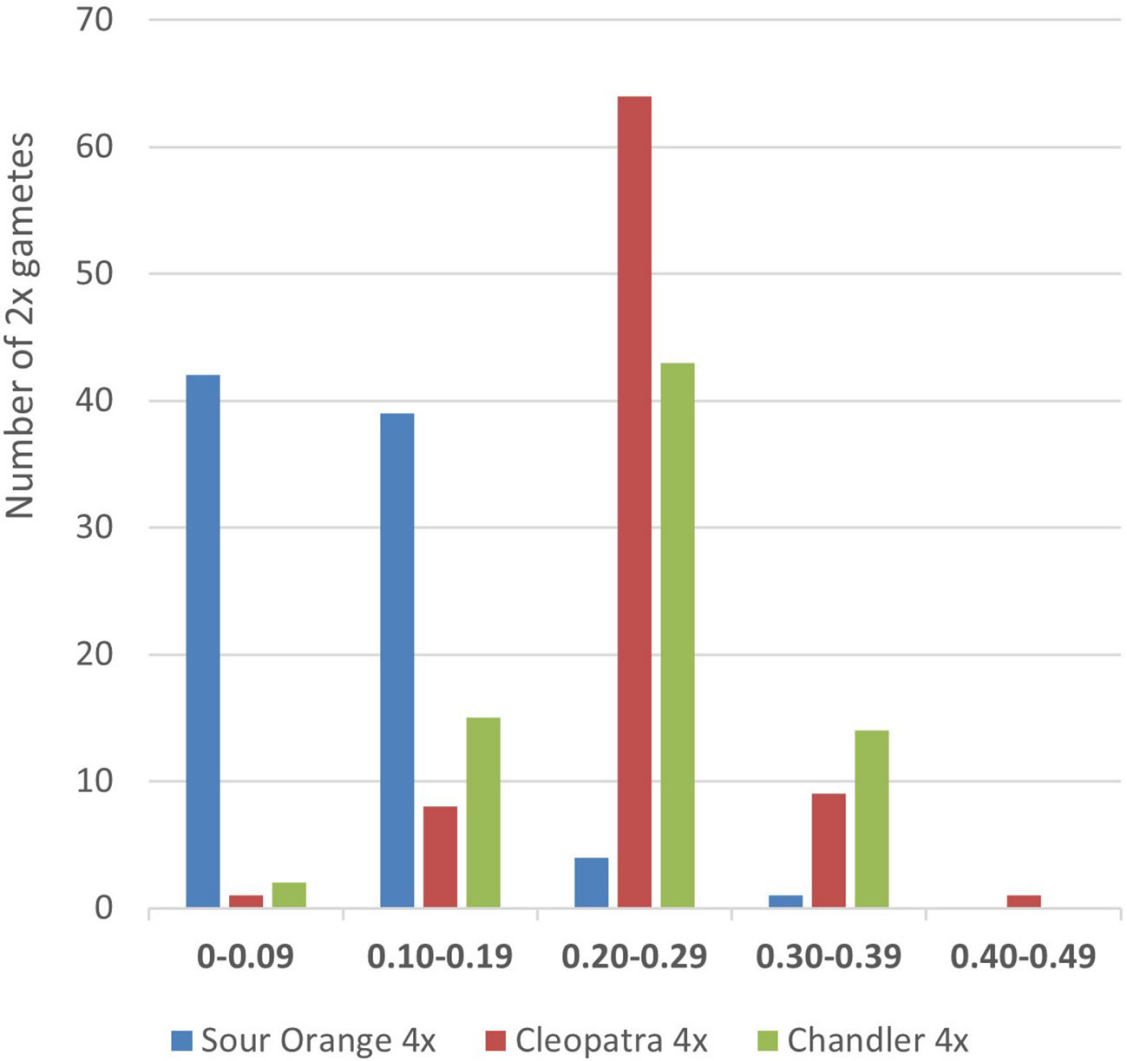
Distribution of genetic distances between tetraploid parents and each gamete obtained by tetraploid ‘Sevillano’ sour orange, ‘Cleopatra’ mandarin, and ‘Chandler’ pummelo.

**Figure 3:**
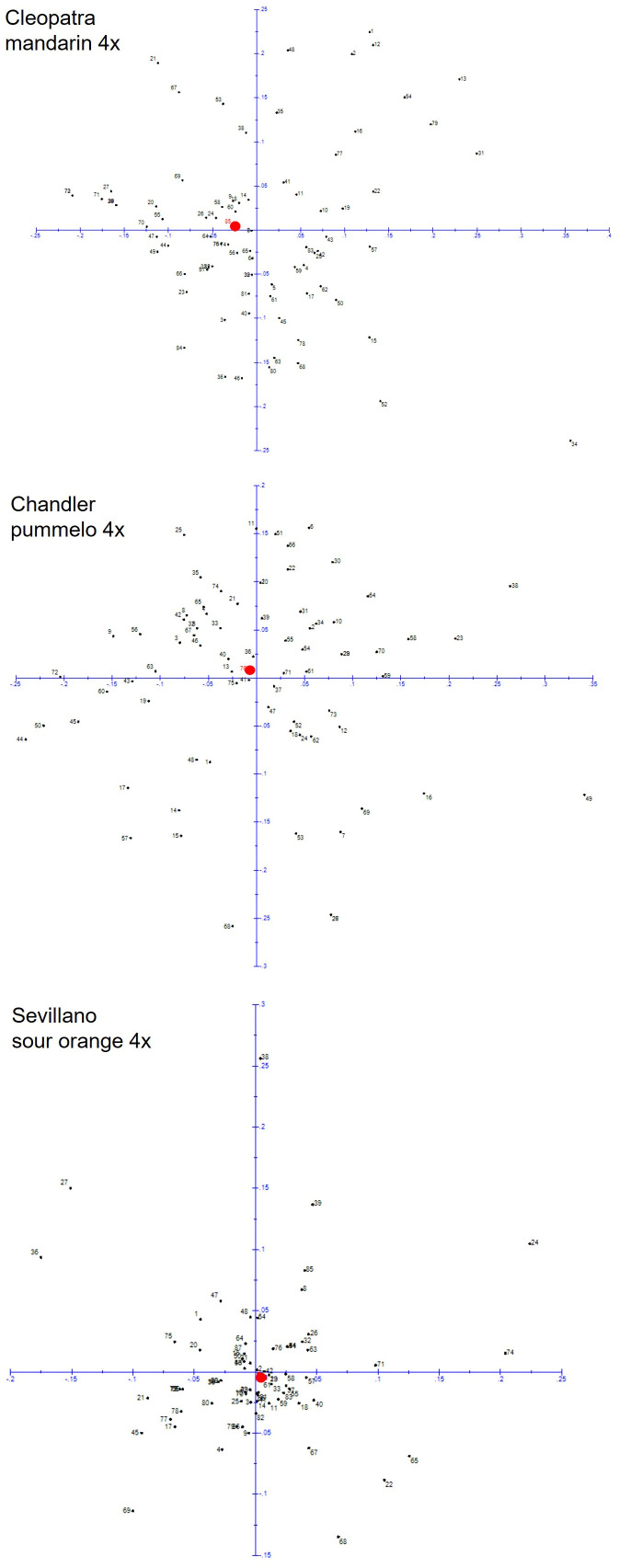
Factorial analysis obtained from the allelic data of diploid gametes produced by the doubled-diploid genotypes ‘Sevillano’ sour orange, ‘Cleopatra’ mandarin, and ‘Chandler’ pummelo. In red is highlighted the doubled diploid parent that originated from diploid gametes.

The average genetic distances between each pair of gametes produced by the tetraploid parents were similar for ‘Cleopatra’ mandarin (0.327 ± 0.003) and ‘Chandler’ pummelo (0.303 ± 0.002) but double that obtained for ‘Sevillano’ sour orange (0.149 ± 0.002) (**Table 2**). The distribution of genetic distances between the tetraploid parents and each diploid gamete is displayed in **Figure 2**. The mode for sour orange measured between 0 and 0.09, whereas for pummelo and mandarin, this measured between 0.20 and 0.29. In addition, ‘Sevillano’ diploid gametes were closer to the original ‘Sevillano’ sour orange diploid cultivar (0.086 ± 0.018) than were the diploid gametes produced by ‘Cleopatra’ mandarin and ‘Chandler’ pummelo (0.215 ± 0.018 and 0.208 ± 0.017, respectively). These results can be observed in the factorial analysis obtained from allelic data with the same number of molecular markers for each chromosome of the three populations of diploid gametes (**Figure 3**). Factorial analysis was drawn on two axes, and ‘Sevillano’ sour orange displayed a more compact dispersion of points closer to the diploid parent than those produced by ‘Chandler’ pummelo and ‘Cleopatra’ mandarin, which were much more dispersed and not as close to the diploid parent.

In previous works, Aleza et al. (2016) and Garavello et al. (2020) performed a genetic analysis of two populations of diploid gametes recovered from tetraploid ‘Clemenules’ clementine and ‘Moncada’ mandarin (*C. clementine* × [*Citrus unshiu* (Mak) Marc. × *Citrus nobilis* Lour.]) as the male parents in 2x × 4x sexual hybridizations. In these studies, the average PHR values for the whole population were 0.649 ± 0.091 and 0.599 ± 0.085, and the average genetic distance between each pair of gametes was 0.291 ± 0.0023 and 0.308 ± 0.0029. These results are similar to those described above for the two populations of diploid gametes produced by ‘Cleopatra’ mandarin and ‘Chandler’ pummelo (**Table 2**). Moreover, the genetic distance values of the whole population of diploid gametes to the diploid parental ‘Clemenules’ clementine (0.176 ± 0.012) and diploid ‘Moncada’ mandarin (0.200 ± 0.093) are much closer to those calculated for ‘Cleopatra’ mandarin (0.215 ± 0.018) and ‘Chandler’ pummelo (0.208 ± 0.017) than the one for ‘Sevillano’ sour orange (0.086 ± 0.018).

### Preferential pairing

Chi-squared analysis of genotypic data for the closest marker to the centromere in each chromosome revealed a significant deviation to the tetrasomic model without DR for all ‘Sevillano’ sour orange chromosomes, except for the mCrCIR04E02 marker of chromosome 3 (**Table 3**). For this tetraploid parent, the deviation was significant for all chromosomes when compared to the theoretical value for the tetrasomic model with maximum equational chromatid segregation. For ‘Cleopatra’ mandarin, unfortunately, no centromeric marker was available for chromosomes 2 and 3. For these two chromosomes, no significant deviation was observed for the full tetrasomic model without DR nor for the tetrasomic model with maximum equational chromatid segregation that can be applied to non-centromeric loci. Among the other chromosomes, a significant deviation to the full tetrasomic model without DR was observed only for the CI07C07 marker of chromosome 7, and it was no more significant for the tetrasomic model with maximum equational chromatid segregation (q-value = 0.0161). No significant deviation to the tetrasomic model without DR was observed for ‘Chandler’ pummelo.

**Table 3:** Estimation of preferential pairing (PP) and double reduction (DR) rate for tetraploid ‘Sevillano’ sour orange, ‘Cleopatra’ mandarin, and ‘Chandler’ pummelo as male parents for markers located close to and far from the centromere within each of the nine chromosomes.

‘Sevillano’ sour orange											
CHR	Locus	DC (cM)	A_1_A_1_	A_1_A_2_	A_2_A_2_	q-Value	SDT	PP	CI	DR	CI
1	1P16721616	1.14	3	74	10	0.0013	S	0.55	0.28–0.76		
*1*	*JK-taa15*	*59.07*	*5*	*74*	*8*					*0.00*	*0–0.31*
2	JK-CAC15	13.63	2	71	14	0.0019	S	0.45	0.16–0.68		
*2*	*JK-TAA41*	*74.99*	*10*	*65*	*12*					*0.19*	*0–0.48*
3	mCrCIR04E02	21.31	9	67	11	0.1292	NS	0.31	0–0.57		
*3*	*MEST256*	*73.57*	*10*	*64*	*13*					*0.08*	*0–0.32*
4	mCrCIR07D06	0.19	3	80	4	0.0000	S	0.76	0.53–0.9		
*4*	*mCrCIR03G05*	*58.92*	*4*	*78*	*5*					*0.15*	*0–0.67*
5	MEST104	17.34	6	72	9	0.0069	*S*	0.49	0.19–0.71		
*5*	*mCrCIR07E12*	*77.79*	*7*	*70*	*10*					*0.08*	*0–0.37*
6	6p4406341	1.40	2	79	6	0.00004	S	0.73	0.48–0.88		
*6*	*mCrCIR01C06*	*82.52*	*7*	*72*	*8*					*0.46*	*0.04–1*
7	mCrCIR03B07	13.04	6	72	9	0.0069	S	0.49	0.19–0.71		
*7*	*FLS-M-313*	*56.43*	*3*	*75*	*9*					*0.00*	*0–0.2*
8	mCrCIR02G02	2.79	5	75	7	0.0013	S	0.59	0.31–0.79		
*8*	*mCrCIR01F04a*	*48.29*	*4*	*78*	*5*					*0.00*	*0–0.22*
9	JI-TCT01	0.64	3	77	7	0.0003	S	0.66	0.4–0.83		
*9*	*Ci02B07*	*52.16*	*4*	*71*	*12*					*0.31*	*0–0.75*
‘Cleopatra’ mandarin											
1	CID0806	5.49	20	43	21	0.04781	NS	0.00	0–0.1		
*1*	*mCrCIR02G08*	*43.94*	*16*	*48*	*20*					*0.145*	*0–0.315*
2	mCrCIR03C08	41.82	12	50	20	0.51187	NS	0.00	0–0.195		
*2*	*JK-TAA41*	*75.07*	*22*	*40*	*22*					*0.285*	*0–0.11*
3	CI08A10	54.29	15	54	15	1.0107	NS	0.00	0–0.25		
*3*	*MEST369*	*70.59*	*7*	*49*	*28*					*0.125*	*0–0.3*
4	CF-CA31	3.93	13	52	19	0.5218	NS	0.00	0–0.205		
*4*	*mCrCIR03G05*	*58.92*	*13*	*54*	*15*					*0.010*	*0–0.185*
5	MEST015	6.90	16	49	19	0.51567	NS	0.00	0–0.16		
*5*	*MEST056*	*87.04*	*14*	*53*	*17*					*0.055*	*0–0.225*
6	MEST132	20.64	16	50	18	0.5218	NS	0.00	0–0.17		
*6*	*CIBE5866*	*93.50*	*25*	*33*	*26*					*0.410*	*0.235–0.57*
7	CI07C07	1.50	11	40	32	5.3E–06	S	0.00	0–0.09		
*7*	*CI02G12*	*72.86*	*14*	*54*	*16*					*0.035*	*0–0.205*
8	mCrCIR07B05	21.09	11	55	18	0.5218	NS	0.00	0–0.28		
*8*	*mCrCIR01F04a*	*48.29*	*9*	*49*	*26*					*0.125*	*0–0.3*
9	MEST308	1.70	17	45	21	0.18644	NS	0.00	0–0.12		
*9*	*MEST187*	*49.24*	*14*	*52*	*17*					*0.060*	*0–0.235*
‘Chandler’ pummelo											
1	1_14053816	3.15	18	51	6	0.24514	NS	0.04	0–0.365		
*1*	*MEST321*	*57.82*	*13*	*47*	*15*					*0.09*	*0–0.28*
2	CX2004	0.11	18	45	12	0.56297	NS	0.00	0–0.195		
*2*	*2_36363713*	*93.26*	*14*	*47*	*14*					*0.06*	*0–0.245*
3	CX0124	19.71	22	46	7	0.04999	NS	0.00	0–0.215		
*3*	*3_51024515*	*101.43*	*16*	*59*	*0*					*0.00*	*0–0.075*
4	mCrCIR07D06	0.19	17	45	13	0.56297	NS	0.00	0–0.195		
*4*	*CIBE3255*	*73.05*	*15*	*47*	*13*					*0.06*	*0–0.245*
5	5_27500244	2.11	13	51	11	1.00781	NS	0.04	0–0.365		
*5*	*MEST056*	*87.04*	*20*	*46*	*9*					*0.11*	*0–0.3*
6	6_5996116	1.13	20	50	5	0.04999	NS	0.00	0–0.33		
*6*	*JK-TAA1*	*87.28*	*14*	*46*	*15*					*0.08*	*0–0.27*
7	7_15708026	8.58	17	46	12	0.56297	NS	0.00	0–0.215		
*7*	*7_20255*	*85.54*	*15*	*42*	*18*					*0.16*	*0–0.35*
8	8_15415145	3.43	8	51	16	0.56297	NS	0.04	0–0.365		
*8*	*mCrCIR02C09*	*40.82*	*17*	*43*	*15*					*0.17*	*0–0.36*
9	MEST308	1.75	13	47	15	0.90603	NS	0.00	0–0.24		
*9*	*9_31288895*	*46.02*	*23*	*38*	*12*					*0.22*	*0.04–0.41*

Allelic configurations for the loci used to estimate DR are highlighted in italics.

LG, linkage group; DC, distance to the centromere in cM [derived from reference genetic map data of Ollitrault et al. (2012a) and location of centromere according to Aleza et al. (2014)]; CHR, chromosome; A_1_A_1_, number of individuals with that allelic configuration; CI, confidence interval; SDT, signification of deviation to tetrasomic model without DR; S, significant; NS, not significant; q-value, p-value of the χ^2^ test for tetrasomy corrected by Benjamini–Hochberg method.

Using likelihood models, for each chromosome, PP values were estimated from the segregation data of the closest marker to the centromere (**Table 3**). The results obtained for doubled diploid ‘Cleopatra’ mandarin and ‘Chandler’ pummelo demonstrated complete tetrasomic inheritance as the best model for both doubled diploid parents and for all chromosomes. Indeed, all chromosomes showed PP values of 0 for ‘Chandler’ pummelo and ‘Cleopatra’ mandarin with only three exceptions: CHR1, CHR5, and CHR8 for ‘Chandler’ pummelo, with PP values very close to 0 (0.04). For doubled diploid ‘Sevillano’ sour orange (**Table 3**), clear intermediate inheritance was the best model for five of the nine chromosomes (CHR1, CHR2, CHR5, CHR7, and CHR8). For chromosome 3, an intermediate inheritance with a tendency toward tetrasomic inheritance (PP = 0.31) was estimated; however, the tetrasomic inheritance without DR was not statistically discarded. CHR4, CHR6, and CHR9, with PP values of 0.76, 0.73, and 0.66, respectively, evidenced an intermediate inheritance with a tendency toward disomy. Variation in PP values among chromosomes has been described in different plant and animal species, such as sugarcane (Jannoo et al., 2004), rose (Bourke et al., 2017), and Pacific oysters (Curole and Hedgecock, 2005). For citrus, variations of PP between chromosomes have already been described in doubled diploid ‘Clemenules’ clementine (Aleza et al., 2016), ‘Moncada’ mandarin hybrid (Garavello et al., 2020), ‘Giant Key’ lime (Ahmed et al., 2020), ‘Mexican’ lime (Rouiss et al., 2018), and two important rootstocks ‘Volkamer’ lemon (*Citrus limonia* Osb.) and ‘Swingle’ citrumelo (*C. paradisi* Macf. × *Poncirus trifoliata* (L.) Raf.) (Calvez et al., 2023). Such differences have also been observed in allotetraploid somatic hybrids between ‘Nova’ tangelo + ‘Hirado Buntan’ pummelo (Xie et al., 2015), ‘Willow leaf’ mandarin + ‘Eureka’ lemon (Kamiri et al., 2011), and ‘Willow leaf’ mandarin + ‘Pomeroy’ *P. trifoliata* L. (Raf.) (Kamiri et al., 2018).

According to Wu et al. (2014); Wu et al. (2018) and Oueslati et al. (2017), the genomes of ‘Cleopatra’ mandarin and ‘Chandler’ pummelo constitute *C. reticulata* and *C. maxima* ancestral species with just one very small introgression of *C. maxima* in CHR3 and *C. reticulata* in CHR2, respectively. The estimation of the genome proportion of these introgressions is respectively 0.5% and 1.9% for ‘Chandler’ pummelo and ‘Cleopatra’ mandarin (Wu et al., 2018). Therefore, doubled-diploid plants of ‘Cleopatra’ mandarin and ‘Chandler’ pummelo can be considered as autotetraploids with four copies of *C. reticulata* and *C. maxima* genomes, respectively. This phylogenomic constitution agrees with the PP values obtained for both genotypes, indicating complete tetrasomic inheritance. Sour orange is an F1 direct hybrid between *C. maxima* and *C. reticulata* (Wu et al., 2014; Oueslati et al., 2017). Thus, the doubled-diploid plant of ‘Sevillano’ sour orange has an interspecific *C. maxima*/*C. reticulata* phylogenomic structure along its entire genome, with two sets of heterologous chromosomes (two sets of *C. maxima* and two sets of *C. reticulata* chromosomes). It is therefore probable that the intermediate model of chromosome segregation that we have observed for the doubled diploid ‘Sevillano’ sour orange results from the genomic differentiation between *C. maxima* and *C. reticulata*. The difference in PP rates between chromosomes suggests variations in the extent of differentiation between the different sets of chromosomes, as proposed by Stebbins (1950).

The present work is the first in citrus to deal with the inheritance pattern of doubled-diploid citrus plants originating from an almost pure ancestral species such as ‘Cleopatra’ mandarin (*C. reticulata*) or ‘Chandler’ pummelo (*C. maxima*). Our results clearly testify to tetrasomic inheritance for these two autotetraploids, as expected. Our results for ‘Sevillano’ sour orange are also the first for a doubled diploid being with interspecific heterozygosity *C. reticulata*/*C. maxima* along the entire genome. Interestingly, despite the full sexual compatibility between *C. maxima* and *C. reticulata* at the diploid level and the high fertility of the diploid ‘Sevillano’ sour orange, the doubled diploid displays intermediate inheritance for the nine chromosomes, with a tendency toward disomy for the three species. Nevertheless, the preferential pairing observed for ‘Sevillano’ sour orange was not found in doubled diploids of ‘Clemenules’ clementine and ‘Moncada’ mandarin, two complex admixtures between *C. maxima* and *C. reticulata*, combining genomic regions with *C. reticulata* homozygosity and *C. reticulata*/*C. maxima* interspecific heterozygosity (Wu et al., 2014; Wu et al., 2018). Indeed, both doubled diploids produced a predominant tetrasomic inheritance pattern (Aleza et al., 2016; Garavello et al., 2020).

The comparison of our results for ‘Sevillano’ sour orange and previous works dealing with other genotypes in interspecific heterozygosity along the entire genome confirms that the preferential pairing rate increases with the phylogenomic distance between ancestral species, as proposed by Rouiss et al. (2018). For instance, doubled diploids of ‘Mexican’ limes displayed predominant disomic inheritance patterns (Rouiss et al., 2018; Ahmed et al., 2020). ‘Mexican’ lime is known to originate from a direct *C. micrantha* × *C. medica* hybridization (Curk et al., 2016; Wu et al., 2018). Whole genome resequencing revealed that the differentiation was higher between these two species than between *C. reticulata* and *C. maxima*; furthermore, the *C. micrantha*/*C. medica* heterozygosity appears to have a greater impact on PP than that between *C. reticulata* and *C. maxima* observed in this work. Similarly, other high PP values published for tetraploid citrus were observed in ‘Swingle’ citrumelo, a citrandarin (Calvez et al., 2020; Calvez et al., 2023), and a somatic hybrid between ‘Willow leaf’ mandarin and ‘Pomeroy’ trifoliate orange (Kamiri et al., 2018). All of these tetraploid parents are in full intergeneric heterozygosity between *P. trifoliata* and *C. reticulata* or *C. maxima* with a higher differentiation between *P. trifoliata* and *Citrus* species than between two *Citrus* species (Wu et al., 2018).

### Double reduction rate

Tetrasomic inheritance is characterized by multivalent formation, which can result in a DR rate. DR can occur due to three major events during meiosis, according to Butruille and Boiteux (2000): i) a crossing-over event between non-sister chromatids, ii) an appropriated disjunction pattern, and iii) subsequent migration of the chromosomal segments carrying a pair of sister alleles to the same gamete. DR may be different between loci, depending on the chromosome on which the locus resides, due to a variability between chromosomes with a tendency toward multivalent formation. The location on the chromosome is also determinant: DR increases toward the telomeres, whereas closer to the centromeres, DR is null (Bourke et al., 2015). The occurrence and frequency of DR impact the segregation ratios of genotypes (Wu et al., 2001). DR was calculated for each chromosome using the molecular marker located furthest from the centromere (**Table 3**). For ‘Sevillano’ sour orange, significant DR was observed in chromosomes 2, 4, 6, and 9. Similarly, chromosomes 1, 2, 3, 6, and 8 displayed significant DR in ‘Cleopatra’ mandarin, whereas for ‘Chandler’ pummelo, the chromosomes with significant DR rates were chromosomes 5, 7, 8, and 9. For most of these chromosomes, the CI included the 1/6 value considered as the maximum possible frequency when quadrivalents are formed and recombinant chromatids migrate to the same pole at anaphase I (Mather, 1936; Gallais, 2003; Stift et al., 2008; Ahmed et al., 2020). Nevertheless, DR estimation was significantly higher in ‘Sevillano’ sour orange for chromosomes 6 and 9, in ‘Cleopatra’ mandarin for chromosomes 2 and 6, and in ‘Chandler’ pummelo for chromosome 9 (**Table 3**). These DR overestimations could be due to negative sporophytic selection, which induces a reduction of heterozygous frequencies for the gene and linked markers (Butruille and Boiteux, 2000). Previous works in other species such as maize and potatoes (Catcheside, 1956; Catcheside, 1959; Welch, 1962; Haynes and Douches, 1993) have also obtained DR rates between 0% and 30%.

## Implications for triploid scion and tetraploid rootstocks breeding programs

Citrus triploid hybrids are useful for scion improvement since they are seedless and bee-friendly, whereas tetraploid rootstocks may display valuable horticultural traits and tolerance to abiotic stresses (Ruiz et al., 2020). Concerning the three tetraploid genotypes analyzed in this study, ‘Chandler’ pummelo is likely to be more useful for scion improvement due to its excellent fruit characteristics, including light pink to very dark pink flesh. ‘Cleopatra’ mandarin and ‘Sevillano’ sour orange are mainly used as rootstocks with no edible fruits for the fresh or juice market, although sour orange is used to make marmalade. Large progenies of triploid hybrids can be obtained by sexual hybridizations using diploid parents producing female unreduced gametes (Aleza et al., 2010a; Cuenca et al., 2011; Cuenca et al., 2015) or by using tetraploid genotypes as male or female parents (Aleza et al., 2012a; Aleza et al., 2012b). Cuenca et al. (2015) demonstrated that second division restitution (SDR) is the main mechanism of female 2n-gamete formation in citrus. The meiotic mechanism originating in the diploid gametes greatly influences the genetic structure of the resulting triploid progeny and must be considered in order to select the most appropriate strategies to recover new triploid hybrids with desired characteristics. Aleza et al. (2016) indicated that SDR-2n gametes transferred relatively low PHR to the progeny (approximately 40% on average), leading to more variable progenies than diploid gametes produced by tetraploid parents, thereby increasing the possibility of obtaining new phenotypes by creating an increasing number of novel multilocus allelic combinations. In this work, we have analyzed the inheritance pattern of doubled diploid ‘Chandler’ pummelo, which showed predominantly tetrasomic segregation with an average of 61.9% PHR value for the whole population and relatively constant values for each chromosome, ranging between 56.9% and 67.5%, as expected for classic tetrasomic segregation (Muller, 1914; Mather, 1936). The rate of 2n ovules is very low in pummelo when compared with other mandarins such as ‘Fortune’ and limits the efficiency of 2x × 2x hybridization for triploid breeding in this horticultural group. Considering that pummelos are non-apomictic varieties, in addition to the tetrasomic inheritance of the tetraploid ‘Chandler’ pummelo, it is an interesting genetic resource to be used as a female parent to recover large populations of triploid pummelo-like hybrids with enough phenotypic diversity for efficient selection.

Due to global climate change and the Huanglongbing crisis (the most important citrus disease worldwide caused by a phloem bacteria), rootstock breeding has become an essential component for the establishment of a sustainable citrus industry all over the world, and tetraploid rootstock breeding is gaining increasing interest. Spontaneous chromosome doubling of nucellar cells is frequently observed in apomictic citrus genotypes producing spontaneous doubled diploid genotypes (Aleza et al., 2010b), whereas in non-apomictic genotypes, chromosome doubling can be induced by using antimitotic chemicals like colchicine or oryzalin (Aleza et al., 2009). Protoplast fusion is another technique used to create tetraploid somatic hybrids, theoretically adding dominant characters of both diploid parental genomes without sexual recombination (Grosser et al., 2000; Ollitrault et al., 2007; Dambier et al., 2011; Grosser and Gmitter, 2011). However, recent studies have revealed some chromosome instability and loss or duplication of substantial genomic regions (Ruiz et al., 2018; Dambier et al., 2022). These methods have been used for citrus rootstock breeding producing a wide range of tetraploid germplasm at intraspecific, interspecific, and even intergeneric levels. The production of sexual tetraploid rootstock hybrids with this tetraploid gene pool has been coined as the ‘tetrazyg’ strategy (Grosser and Gmitter, 2011), and the efficiency of this strategy depends on the inheritance pattern of the tetraploid hybrids used as parents. Recently, Calvez et al. (2020); Calvez et al. (2023) stated that PP influences the transmission of PHR, reducing effective interspecific recombination and, thus, the genetic and phenotypic diversity of the hybrid population recovered from intergeneric *Citrus* × *Poncirus* tetraploid hybrids. The highly disomic inheritance pattern observed in doubled diploid citrandarin and ‘4475’ and ‘Swingle’ citrumelos allows for the retention of many genes of interest from the original intergeneric diploid parents, which is of great importance for preventing the overall breakage of the favorable complex multilocus genotypic structure selected at the diploid level (Calvez et al., 2020; Calvez et al., 2023). In interspecific and intergeneric hybrids, a disomic tendency is therefore favorable for rootstock breeding with the view to transmitting by the diploid gamete a large part of the genetic value selected in the elite diploid rootstock.

In this work, we analyzed the meiotic behavior of two doubled diploids derived from two important diploid rootstocks. Sour orange was the most used rootstock worldwide during the first half of the 20th century. It is highly resistant to phytophthora, is tolerant to the nematode, displays very good adaptation to most soil types, has the ability to induce fruits of high quality in the grafted variety, and has tolerance to many abiotic factors (Castle, 2010). However, citrus tristeza virus (CTV) induces the decline and death of citrus varieties grafted onto sour orange rootstock due to a hypersensitive response to CTV of the sour orange cells below the bud union (Gómez-Muñoz et al., 2017). The CTV crisis killed millions of trees grafted onto sour orange (Moreno and Garnsey, 2010; Lee and Keremane, 2013) and resulted in an important diversification of citrus rootstocks. ‘Cleopatra’ mandarin is one of the citrus rootstocks most tolerant to salt and high alkalinity stresses. It provides good fruit quality and is suitable for shallow soils. However, it is sensitive to phytophthora, nematodes, and waterlogging (Castle, 2010). It is a good parent for rootstock breeding at the diploid level. We observed contrasted meiotic behavior for ‘Cleopatra’ mandarin and ‘Sevillano’ sour orange doubled diploid plants in direct relation with their interspecific versus intraspecific origin. For the tetraploid ‘Cleopatra’ mandarin (four copies of *C. reticulata*), a clear tetrasomic inheritance pattern has been identified, with non-significant PP values. This tetrasomic inheritance leads to higher levels of recombination and potential segregation than disomy. However, ‘Cleopatra’ mandarin is part of the rootstock with the lower heterozygosity; therefore, the variability of the diploid gamete is still limited, and it is expected that a significant part of the diploid ‘Cleopatra’ genetic value is transmitted by the diploid gametes. In any case, with an average PHR of 58.3%, this part of the genetic value should be higher than that transmitted by the haploid gametes produced by the diploid ‘Cleopatra’ mandarin. ‘Sevillano’ sour orange, the F1 hybrid between *C. reticulata* × *C. maxima*, displayed a mostly intermediate inheritance pattern, with a tendency toward disomy with PP values approximately 0.5 to 0.7. This meiotic behavior is similar to the one described by Calvez et al. (2023) for ‘Volkamer’ lemon (F1 interspecific hybrid *C. reticulata* × *C. medica*). With an average PHR of 83.7%, the tetraploid sour orange transmits, by its diploid gametes, a large part of the genetic value of the original diploid sour orange. However, the intermediate PP allows for interspecific genetic recombination leading to a partial retention of the entire set of genes from the original diploid ‘Sevillano’ sour orange. This opens the way for the counter-selection of unfavorable genes such as those involved in the hypersensitive response to CTV (Gómez-Muñoz et al., 2017).

## Conclusions

In this work, we have studied the inheritance pattern of three doubled diploid genotypes, ‘Chandler’ pummelo, ‘Cleopatra’ mandarin, and ‘Sevillano’ sour orange, with SSR and SNP markers distributed in the nine citrus chromosomes. The estimation of allele dosage of *C. maxima* and *C. reticulata* in each triploid progeny recovered in 2x × 4x crosses with clementine as the female parent and the three doubled diploid genotypes as the male parents allowed us to calculate PHR, as well as to estimate PP and the genotypic variability of the diploid gamete progenies. The ‘Chandler’ pummelo and ‘Cleopatra’ mandarin showed clear tetrasomic inheritance patterns with insignificant PP values for all chromosomes, while the sour orange displayed an intermediate inheritance pattern with a tendency toward disomy for three chromosomes, with PP values varying from 0.5 to 0.7. These results clearly demonstrate the impact of the phylogenomic origin on inheritance mode and PHR. In doubled diploid genotypes originating from a single species (i.e., autotetraploid) such as ‘Chandler’ pummelo (*C. maxima*) or ‘Cleopatra’ mandarin (*C. reticulata*), the tetrasomic inheritance, theoretically favoring tetravalent formation during meiosis, led to PHR values between 55% and 66%, depending on the DR rate. In contrast, the intermediate inheritance of the allotetraploid *C. maxima*/*C. reticulata* ‘Sevillano’ sour orange resulted in higher values of PHR (from 0.77 to 0.91, according to the chromosomes). Interestingly, while there is complete sexual compatibility between *C. maxima* and *C. reticulata* with full fertility of F1 interspecific hybrids at the diploid level, the genome divergence between the two species results in preferential chromosome pairing in tetraploid plants. The molecular determinants of this meiotic behavior remain to be discovered. The tetraploid ‘Chandler’ pummelo should be an interesting female parent to create large progenies of pummelo-like triploid hybrids. The intermediate inheritance of the ‘Sevillano’ sour orange results in the transmission by the diploid gamete of a significant part of the genetic value of the original diploid sour orange gamete and allows for the interspecific recombination and potential elimination of unfavorable genes. The tetraploid ‘Sevillano’ sour orange is therefore a promising genetic resource for developing new rootstock combining most of the interesting traits of sour orange with the tolerance to CTV. Despite tetrasomic inheritance, the low level of heterozygosity of ‘Cleopatra’ mandarin makes the doubled diploid promising for creating tetraploid rootstock with a good level of inheritance of the dominant favorable traits of this genotype.

## Data availability statement

All relevant data is contained within the article: The original contributions presented in the study are included in the **Supplementary Material** (https://www.frontiersin.org/articles/10.3389/fpls.2023.1327872/full#supplementary-material), further inquiries can be directed to the corresponding authors.

## Author contributions

PA: Conceptualization, Formal analysis, Funding acquisition, Project administration, Supervision, Validation, Writing – original draft, Writing – review & editing, Methodology. MG: Data curation, Writing – original draft. HR: Data curation, Writing – original draft. AB: Data curation, Writing – original draft. AG: Data curation, Writing – original draft. MH: Data curation, Writing – original draft. LN: Writing – review & editing. PO: Conceptualization, Formal analysis, Methodology, Software, Supervision, Validation, Writing – review & editing.
