# Supplementary material for: Inheritance pattern of tetraploids pummelo, mandarin, and their interspecific hybrid sour orange is highly influenced by their phylogenomic structure
Source: Front Plant Sci. 2023 Dec 8;14:1327872. doi: 10.3389/fpls.2023.1327872 (PMC10739408; doi:10.3389/fpls.2023.1327872)
Supplement: Supplementary file 6 [file DataSheet_1.docx]

Ahmad, R., Struss, D., and Southwick, S.M. (2003). Development and characterization of microsatellite markers in Citrus. J. Am. Soc. Hortic. Sci. 128, 584–590. doi.org/10.21273/JASHS.128.4.0584.

Aleza, P., Froelicher, Y., Schwarz, S., Agustí, M., Hernández, M., Juárez, J., Luro, F., Morillon, R., Navarro, L., Ollitrault, P. (2011). Tetraploidization events by chromosome doubling of nucellar cells are frequent in apomictic citrus and are dependent on genotype and environment. Ann. Bot, 108(1):37–50. Doi: 10.1093/aob/mcr099.

Chen, C., Bowman, K.D., Choi, Y.A., Dang, P.M., Rao, M., Huang, S., Soneji, J.R., Mccollum, T.G., Gmitter, Jr, F.G. (2007). EST-SSR genetic maps for Citrus sinensis and Poncirus trifoliata. Tree Genet. Genomes. 4:1-10

Cuenca, J., Froelicher, Y., Aleza, P., Juarez, J., Navarro, L., Ollitrault, P. (2011). Multilocus half-tetrad analysis and centromere mapping in citrus: Evidence of SDR mechanism for 2nmegagametophyte production and partial chiasma interference in mandarin cv 'Fortune'. Heredity 107: 462-470. Doi: 10.1038/hdy.2011.33.

Curk, F., Ancillo, G., Ollitrault, F., Perrier, X., Jacquemoud-Collet, J.P., Garcia-Lor, A., Navarro, L., Ollitrault, P. (2015). Nuclear species-diagnostic SNP markers mined from 454 amplicon sequencing reveal admixture genomic structure of modern citrus varieties. PLoS One 10(5):e0125628. doi:10.1371/journal.pone.0125628.eCollection

Froelicher, Y., Dambier, D., Bassene, J.B., Costantino, G., Lotfy, S., Didout, C., Beaumont, V., Brottier, P., Risterucci, A.M., Luro, F., Ollitrault, P. (2008). Characterization of microsatellite markers in mandarin orange (Citrus reticulata Blanco). Mol. Ecol. Resour. 8(1):119–122. doi: 10.1111/j.1471-8286.2007.01893.x.

Garcia-Lor, A., Luro, F., Navarro, L., Ollitrault, P. (2012). Comparative use of InDel and SSR markers in deciphering the interspecific structure of cultivated citrus genetic diversity: a perspective for genetic association studies. Mol Genet Genomics. 287(1):77–94. doi: 10.1007/s00438-011-0658-4.

Garcia-Lor, A., Curk, F., Snoussi-Trifa, H., Morillon, R., Ancillo, G., Luro, F., Navarro, L., Ollitrault, P. (2013). A nuclear phylogenetic analysis: SNPs, indels and SSRs deliver new insights into the relationships in the ‘true citrus fruit trees’ group (Citrinae, Rutaceae) and the origin of cultivated species. Ann. Bot. 111: 1-19. doi: 10.1093/aob/mcs227.

Kamiri, M., Stift, M., Srairi, I., Costantino, G., El Moussadik, A., Hmyene, A., Bakry, F., Ollitrault, P., Froelicher, Y. (2011). Evidence for non-disomic inheritance in a Citrus interspecific tetraploid somatic hybrid between C. reticulata and C. limon using SSR markers and cytogenetic analysis. Plant Cell Rep. 30(8):1415–1425. Doi: 10.1007/s00299-011-1050-x.

Kijas, J.M.H., Thomas, M.R., Fowler, J.C.S., Roose, M.L. (1997). Integration of trinucleotide microsatellites into a linkage map of Citrus. Theor Appl Genet. 94(5):701–706. doi.org/10.1007/s001220050468.

Luro, F.L., Costantino, G., Terol, J., Argout, X., Allario, T., Wincker, P., Talon, M., Ollitrault, P., Morillon, R. (2008). Transferability of the EST-SSRs developed on Nules clementine (Citrus clementina Hort ex Tan) to other Citrus species and their effectiveness for genetic mapping. BMC Genom. 9(1), 287. doi.org/10.1186/1471-2164-9-287.

Ollitrault, F., Terol, J., Pina, J.A., Navarro, L., Talon, M., Ollitrault, P. (2010). Development of SSR markers from Citrus clementina (Rutaceae) BAC end sequences and interspecific transferability in Citrus. Am J Bot, 97(11):e124–e129. doi: 10.3732/ajb.1000280.

Ollitrault, P., Terol, J., Garcia-Lor, A., Berard, A., Chauveau, A., Froelicher, Y., Belzile, C., Morillon, R., Navarro, L., Brunel, D., Talon, M. (2012a). SNP mining in C. clementina BAC end sequences; transferability in the Citrus genus (Rutaceae), phylogenetic inferences and perspectives for genetic mapping. BMC Genom. 13:13. doi: 10.1186/1471-2164-13-13.

Ollitrault, P., Terol, J., Chen, C., Federici, C., Lotfy, S., Hippolyte, I., Ollitrault, F., Bérard, A., Chauveau, A., Cuenca, J., Costantino, G., Kacar, Y., Mu, L., Garcia-Lor, A., Froelicher, Y., Aleza, P., Boland, A., Billot, C., Navarro, L., Luro, F., Roose, M.L., Gmitter, F.G., Talon, M., Brunel, D. (2012b). A reference genetic map of C. clementine Hort. ex Tan.; citrus evolution inferences from comparative mapping. BMC Genom. 13: 593. doi.org/10.1186/1471-2164-13-593.

Ollitrault, F., Terol, J., Alonso, A., Martin, A., Pina, J.A., Navarro, L, Talon, M., Ollitrault, P. (2012c). Development of InDel markers from Citrus clementina (Rutaceae) BAC-end sequences and interspecific transferability in Citrus. Am. J. Bot. 99(7):e268–e273. doi: 10.3732/ajb.1100569.
